# Predicting burnout in radiology nurses: an interpretable machine learning model developed and externally validated in a multi-center study

**DOI:** 10.3389/fpubh.2026.1914779

**Published:** 2026-08-11

**Authors:** Linlin Guo, Qian Zhang, Luxin Sun, Peiyao Hu, Zhaorui Wang, Jianhua Gu, Qian Sun, Youfu He

**Affiliations:** 1 The Second Department of Radiotherapy, The First Affiliated Hospital of Zhengzhou University, Zhengzhou, Henan, China; 2 Department of Radiology, The First Affiliated Hospital of Zhengzhou University, Zhengzhou, Henan, China; 3 Office of Scientific Research, The Fifth Clinical Medical College of Henan University of Chinese Medicine (Zhengzhou People’s Hospital), Zhengzhou, Henan, China; 4 Translational Medicine Research Center, The Fifth Clinical Medical College of Henan University of Chinese Medicine (Zhengzhou People’s Hospital), Zhengzhou, Henan, China; 5 Department of Emergency Medicine, Qilu Hospital of Shandong University, Jinan, Shandong, China; 6 Department of Radiology, Qilu Hospital of Shandong University (Qingdao), Qingdao, Shandong, China; 7 Department of Cardiology, Guizhou Provincial People’s Hospital, Guiyang, Guizhou, China

**Keywords:** burnout, machine learning, predictive modeling, radiology nurses, SHAP, XGBoost

## Abstract

**Objective:**

To develop and externally validate an interpretable machine learning model for predicting individual burnout risk among radiology nurses, and to identify modifiable, non-linear risk thresholds to guide precision prevention.

**Methods:**

A two-center, two-stage design was used. A development cohort (*n* = 219) from six tertiary hospitals in China trained an XGBoost model. An independent external validation cohort (*n* = 234) was collected 1.5 years later from two tertiary centers (one partially overlapping with the development sites but with entirely independent participants) to rigorously test generalizability. Feature selection used bootstrapped LASSO with permutation testing. Model performance was assessed by discrimination, calibration, and decision curve analysis. SHAP (SHapley Additive exPlanations) provided global and individual-level interpretability.

**Results:**

The XGBoost model achieved strong discrimination in external validation (AUC = 0.859). In the internal test set, XGBoost outperformed logistic regression and other machine learning algorithms (AUC: 0.963 vs. 0.910 for logistic regression). SHAP analysis identified Effort-Reward Imbalance and Overcommitment as dominant predictors, and revealed a potential inflection point at approximately 65 weekly work hours, beyond which burnout risk escalated disproportionately in the SHAP analysis. Decision curve analysis confirmed net clinical benefit across threshold probabilities of 5 –65%. Individualized risk profiles were generated for actionable nurse management.

**Conclusion:**

This externally validated, interpretable XGBoost-SHAP framework moves beyond population-averaged associations. By uncovering specific non-linear thresholds (e.g., 65 h/week workload) and providing personalized risk profiles, it enables nurse managers to shift from reactive crisis management to data-driven, precision prevention of burnout in radiology nurses.

## 1 Introduction

Workforce burnout has escalated into a systemic crisis within global healthcare, widely recognized not merely as an occupational hazard but as a fundamental threat to patient safety and care quality (1–3). Radiology nurses operate within a distinct, high-stakes ecological niche, navigating rapid patient turnover, cumulative radiation exposure risks, and the cognitive load of managing sophisticated interventional technologies (4, 5). As diagnostic imaging becomes central to modern medicine on increasing workload, the resultant chronic strain has made this subpopulation disproportionately vulnerable to exhaustion and attrition (6).

While theoretical frameworks such as Effort-Reward Imbalance (ERI) have been extensively documented (7–9), existing studies have primarily relied on conventional linear statistical models to describe population-level associations (10, 11). These approaches inherently oversimplify the complex, non-linear reality of human psychology—for instance, the impact of working hours on mental health may exhibit a “tipping point” beyond which resilience collapses rapidly, a nuance that linear models fail to capture (12).

Machine learning (ML) offers a promising approach, shifting the paradigm from explanatory epidemiology to predictive precision medicine (12). Advanced algorithms, such as Extreme Gradient Boosting (XGBoost), can process high-dimensional interactions to forecast outcomes with superior accuracy (13, 14). However, clinical implementation is often hindered by the “black box” problem, where opaque algorithmic decisions limit trust and actionable guidance (15, 16).

To address these limitations, this study applies an interpretable ML framework to multi-center datasets of radiology nurses. Our objectives were to: (1) develop and externally validate a robust model predicting individual burnout risk; (2) leverage SHapley Additive exPlanations (SHAP) to reveal non-linear dose–response relationships and critical stress thresholds; and (3) provide granular, individualized risk profiles to facilitate tailored, data-driven preventive strategies.

## 2 Methods

### 2.1 Study design and population

This study adopted a two-stage design comprising multi-center model development and independent external validation.

*Development cohort*: The development cohort data were derived from a previous cross-sectional investigation conducted between January and March 2024. This dataset comprised 219 frontline radiology nurses recruited via convenience sampling from six major tertiary hospitals in China, including Qilu Hospital of Shandong University (Qingdao), Affiliated Hospital of Jining Medical University, The First Affiliated Hospital of Zhengzhou University, West China Hospital of Sichuan University, The First Affiliated Hospital of Chongqing University, and Peking University First Hospital.

*External validation cohort*: An independent cohort was recruited in December 2025 from two tertiary centers: The First Affiliated Hospital of Zhengzhou University and Guizhou Provincial People’s Hospital. Notably, while The First Affiliated Hospital of Zhengzhou University also contributed to the development cohort, the external validation participants were recruited 1.5 years later (December 2025 vs. January–March 2024) from different departmental units and shift teams, ensuring no participant overlap between the two cohorts. Thus, this validation design is best characterized as temporally independent with partial geographic overlap, rather than purely temporally independent. The validation set comprised 234 participants in total. All participants met identical inclusion and exclusion criteria to the development cohort.

The study included radiology nurses from radiotherapy, interventional radiology, diagnostic imaging, and general radiology settings. All participants were analyzed as a single cohort; subgroup analyses by specialty were not pre-specified. To eliminate any potential confounding from site overlap, we verified that no individual nurse participated in both cohorts by cross-checking unique employee identifiers, which were anonymized prior to analysis.

All participants provided written informed consent. The study was conducted and reported in accordance with the TRIPOD statement for type 2b studies (development and validation using separate data).

### 2.2 Outcome definition and candidate predictors

The primary outcome was a binary variable representing high burnout risk, defined as a composite score of ≥3.5 on the Maslach Burnout Inventory–General Survey (MBI-GS). This instrument, validated for Chinese healthcare professionals, quantifies burnout across three dimensions: emotional exhaustion, depersonalization, and personal accomplishment. The officially licensed Chinese-Mandarin translation was obtained from Mind Garden, Inc.,^1^ and the scale demonstrated high reliability across cohorts (Cronbach’s *α* = 0.82–0.85). The composite score was calculated using the validated weighted formula (0.4 × emotional exhaustion + 0.3 × depersonalization + 0.3 × [6 − personal accomplishment]), with the ≥3.5 threshold established to identify moderate-to-severe burnout levels requiring intervention according to the classification system of Kalimo et al. (17)

Twenty-two candidate predictors were considered, encompassing demographic variables (age, sex, professional title, educational attainment, tenure, marital status, chronic disease, and caregiver responsibilities). Caregiver responsibilities included older adult care and childcare, each coded as binary variables (0 = no, 1 = yes). Occupational characteristics included weekly work hours, overtime frequency, and shift work. To capture psychosocial job demands, we incorporated the five subscale scores from the Chinese Nurse Stressor Scale (CNSS): stress from nursing profession and work, time allocation and workload, work environment and resources, patient care challenges, and management and interpersonal relationships. Similarly, to model the effort-reward imbalance construct, the three core components from the Effort-Reward Imbalance (ERI) scale—effort, reward, and overcommitment—were included as separate continuous predictors. Categorical variables were encoded using one-hot (dummy) encoding, with the reference level set to the most frequent category. Data completeness was high across all variables (<2% missing). For XGBoost, missing values were handled natively via the algorithm’s sparsity-aware split-finding procedure; for Logistic Regression, SVM, and Random Forest, median imputation was used for continuous variables and mode imputation for categorical variables, applied using training-set statistics only.

### 2.3 Feature selection and data partitioning

We implemented stability-driven variable selection using LASSO penalized logistic regression across 1,000 bootstrap resamples (18), benchmarked against a null distribution via Permutation Stability Testing. Only predictors with selection frequency significantly exceeding the 95th percentile (permutation *p* < 0.05) were retained. Importantly, this feature selection procedure was performed exclusively on the training set (see below); the internal test set was completely sequestered and not accessed during this step.

The development cohort was first partitioned into a training set (*n* = 152, 70%) and an internal test set (*n* = 67, 30%) using stratified sampling based on burnout status. The internal test set was then completely sequestered and was not accessed during any feature selection, hyperparameter tuning, or model development steps. The external validation cohort (*n* = 234) was isolated from the outset and remained untouched until the final model evaluation.

### 2.4 Model development and evaluation

*Modeling pipeline*: To ensure methodological transparency and prevent data leakage, all modeling procedures were performed following a strict chronological pipeline: (1) data split—the development cohort was partitioned into training and internal test sets; (2) feature selection—performed exclusively on the training set using bootstrapped LASSO with permutation testing; (3) hyperparameter tuning—conducted solely within the training set via 5-fold cross-validation; (4) model fitting—final models were trained on the full training set using selected features and optimized hyperparameters; (5) internal testing—models were evaluated on the held-out internal test set; and (6) external validation—the finalized XGBoost model was applied to the independent external validation cohort without any re-training or re-calibration. At no point during feature selection, hyperparameter tuning, or model training were the internal test set or external validation cohort used to inform model decisions. Four supervised machine learning algorithms were trained on the selected parsimonious feature set using the training partition of the development cohort. These included a regularized logistic regression model (benchmark), Random Forest (RF), Support Vector Machine (SVM) with a radial basis function kernel, and Extreme Gradient Boosting (XGBoost).

Hyperparameters for each algorithm were systematically optimized within the training set using a 5-fold cross-validation scheme, guided by maximizing the mean area under the receiver operating characteristic (ROC) curve (AUC). The hyperparameter search spaces and optimal values for each algorithm are detailed in Supplementary Table S1. The predictive performance of the finalized models was then evaluated in a hierarchical manner: first on the internal hold-out test set, and subsequently on the external validation cohort. The external validation was conducted without any re-calibration or re-training to rigorously assess generalizability in a truly independent setting. XGBoost predicted probabilities were generated via the native sigmoid transformation of raw scores, using the default 0.5 classification threshold. No post-hoc calibration was applied.

Prior to model fitting, continuous predictors were standardized to mean = 0 and standard deviation = 1 for Logistic Regression and SVM (using training-set means and SDs, with the same parameters applied to test and validation sets). Tree-based methods (Random Forest and XGBoost) used raw values and do not require scaling.

To address class imbalance (38.8% burnout prevalence in the training set), XGBoost used scale_pos_weight = 1.58 (ratio of negative to positive samples); Logistic Regression and SVM used class_weight = “balanced”; Random Forest used class_weight = “balanced.”

All classification metrics (sensitivity, specificity, precision, F1-score, accuracy) were calculated using the default probability threshold of 0.5.

All 95% confidence intervals were derived from 2,000 participant-level bootstrap replicates (resampling individual participants with replacement, preserving the original sample size of each cohort).

Discriminative ability was quantified using the AUC, accuracy, sensitivity (recall), specificity, precision (positive predictive value), and the F1-score. To assess the clinical utility and generalizability of the models, we scrutinized model calibration via calibration plots and Brier scores in both internal and external datasets. Calibration was further quantified using the calibration intercept and calibration slope, with values of 0 and 1 indicating perfect calibration, respectively. Furthermore, Decision Curve Analysis (DCA) was performed on the external validation set to estimate the net benefit of the model across a range of threshold probabilities, comparing it against default strategies of “treating all” or “treating none.”

### 2.5 Model interpretability using SHAP

To move beyond “black box” predictions and provide transparent, clinically actionable insights, the best-performing model was subjected to a comprehensive post-hoc interpretability analysis using SHAP. This game-theoretic framework attributes a precise contribution (a SHAP value) to each feature for every individual prediction, ensuring both local accuracy and global consistency. Global feature importance was visualized by plotting the mean absolute SHAP values for each predictor. To elucidate the directionality and non-linear complexity of feature effects, SHAP dependence plots were generated for the most influential variables. Finally, to deconstruct the model’s decision-making logic at a granular, individual level, SHAP waterfall plots were created for prototypical high-risk and low-risk cases, illustrating exactly how the contributions from different predictors aggregate to form the final risk prediction. SHAP values were computed on the internal test set using the finalized XGBoost model trained on the full training set. The internal test set was chosen for interpretability because it represents unseen data from the same source population, enabling clear visualization of individual-level contributions; external validation was reserved for assessing overall generalizability without over-interpreting feature-level patterns in a single independent sample.

### 2.6 Sample size considerations

The development cohort comprised 219 participants, including 85 participants with high burnout (38.8%). No formal sample-size calculation specific to XGBoost was available; therefore, sample-size adequacy was assessed using the events-per-variable (EPV) framework as a pragmatic reference. Although the conventional threshold of 10 EPV was developed for regression models and does not directly apply to ensemble machine-learning methods, a more conservative threshold of 15 EPV was considered. Based on the five predictors retained by the stability-based feature-selection procedure—overcommitment, effort, weekly working hours, older adult care responsibility, and professional work stress—the effective EPV was 85/5 = 17, which meets this conservative benchmark for the final selected predictors. However, because the full modeling process included 22 candidate variables, this EPV estimate should be interpreted with caution.

The initial candidate set comprised 22 variables. Because predictor selection was data-driven, the EPV calculation should be interpreted as an approximate assessment rather than a formal guarantee against overfitting. Model transportability was therefore evaluated in an independent external cohort recruited 1.5 years later from different hospital units (*n* = 234); this cohort was not used for model fitting, retraining, or recalibration.

### 2.7 Sensitivity analyses and software

Two sensitivity analyses were conducted: (1) substituting ERI components with the traditional ratio; (2) excluding participants with chronic diseases. All statistical analyses and machine learning models were implemented in R version 4.3.1, utilizing the VSOLassoBag, caret, xgboost, and shapr packages. Additionally, a sensitivity analysis using an alternative burnout definition based on dimensional cutoffs (emotional exhaustion > 12, depersonalization > 7, reduced personal accomplishment > 18; high burnout defined as ≥2 dimensions) was conducted, with results presented in Supplementary Table S2.

## 3 Results

### 3.1 Participant characteristics and prevalence of burnout

The study included a total of 453 radiology nurses, comprising a development cohort (*n* = 219) characteristic of the initial study phase, and an independent external validation cohort (*n* = 234)—temporally independent with partial geographic overlap—introduced to rigorously test model generalizability. The prevalence of high occupational burnout (MBI-GS score ≥3.5) showed notable variation across settings: 38.8% (85/219) in the development cohort versus 29.1% (68/234) in the external validation cohort. Baseline characteristics stratified by cohort and burnout status are summarized in Table 1. Consistent patterns emerged: high-burnout nurses reported longer weekly work hours (*p* < 0.05) and pronounced effort-reward imbalance (higher Effort and Overcommitment, lower Reward; all *p* < 0.05). Demographic factors showed limited associations, whereas work-related stressors remained robust risk markers across settings.

**Table 1:** Baseline characteristics of radiology nurses in the training, testing, and independent external validation cohorts, stratified by burnout status.

Variable	Training set (*n* = 152)			Testing set (*n* = 67)			Ext. validation set (*n* = 234)		
	Non-burnout (*n* = 93)	High burnout (*n* = 59)	*p*	Non-burnout (*n* = 41)	High burnout (*n* = 26)	*p*	Non-burnout (*n* = 166)	High burnout (*n* = 68)	*p*
Demographics									
Age (years), mean (SD)	26.59 (4.76)	26.63 (4.39)	0.963	25.83 (4.78)	26.69 (4.58)	0.467	32.11 (6.48)	29.06 (5.60)	0.001
Gender, *n* (%)									
Marital status, *n* (%)			0.193			0.591			0.181
Unmarried	30 (32.3)	17 (28.8)		9 (22.0)	5 (19.2)		64 (38.6)	35 (51.5)	
Married	63 (67.7)	40 (67.8)		31 (75.6)	19 (73.1)		100 (60.2)	32 (47.1)	
Divorced/other	0 (0.0)	2 (3.4)		1 (2.4)	2 (7.7)		2 (1.2)	1 (1.5)	
Education Level, *n* (%)			0.823			0.878			0.002
Associate degree	30 (32.3)	17 (28.8)		19 (46.3)	11 (42.3)		4 (2.4)	7 (10.3)	
Bachelor’s degree	52 (55.9)	36 (61.0)		18 (43.9)	13 (50.0)		152 (91.6)	51 (75.0)	
Master’s or higher	11 (11.8)	6 (10.2)		4 (9.8)	2 (7.7)		10 (6.0)	10 (14.7)	
Occupational characteristics									
Years of experience	2.73 (3.60)^a^	3.14 (3.21)^a^	0.483	1.66 (2.94)^a^	2.69 (3.03)^a^	0.17	5.00 [3.00, 9.75]^b^	3.00 [2.00, 6.25]^b^	0.002
Professional title, *n* (%)			0.022			0.376			0.061
Nurse/Snr. nurse	25 (26.9)	10 (16.9)		16 (39.0)	8 (30.8)		94 (56.6)	47 (69.1)	
Nurse practitioner	33 (35.5)	29 (49.2)		13 (31.7)	10 (38.5)		65 (39.2)	16 (23.5)	
Snr. practitioner	34 (36.6)	15 (25.4)		11 (26.8)	5 (19.2)		7 (4.2)	5 (7.4)	
Chief nurse	1 (1.1)	5 (8.5)		1 (2.4)	3 (11.5)		–	–	
Weekly work hours, mean (SD)	59.95 (6.02)	66.00 (7.74)	<0.001	61.37 (6.61)	65.50 (7.72)	0.023	47.47 (15.49)	42.29 (18.91)	0.031
Overtime freq., *n* (%)			<0.001			0.492			<0.001
Rare/<5 h/week	33 (35.5)	4 (6.8)		12 (29.3)	6 (23.1)		100 (60.2)	20 (29.4)	
5–10 h/week	27 (29.0)	18 (30.5)		16 (39.0)	8 (30.8)		52 (31.3)	30 (44.1)	
10 h/week	33 (35.5)	37 (62.7)		13 (31.7)	12 (46.2)		14 (8.4)	18 (26.5)	
Health and family									
Chronic disease, *n* (%)	1 (1.1)	5 (8.5)	0.063	1 (2.4)	3 (11.5)	0.316	16 (9.6)	9 (13.2)	0.565
Older adult care stress, *n* (%)	17 (18.3)	19 (32.2)	0.076	9 (22.0)	4 (15.4)	0.73	89 (53.6)	34 (50.0)	0.72
Childcare stress, *n* (%)	50 (53.8)	40 (67.8)	0.122	22 (53.7)	13 (50.0)	0.967	86 (51.8)	30 (44.1)	0.355
Psychosocial stressors^c^									
Professional and work	11.02 (3.09)	12.83 (2.85)	<0.001	10.46 (2.66)	12.50 (2.76)	0.004	15.63 (5.43)	22.57 (3.30)	<0.001
Time management	11.60 (2.49)	12.19 (3.51)	0.232	11.29 (2.67)	11.46 (2.42)	0.794	15.35 (5.75)	22.84 (2.92)	<0.001
Work environment	10.85 (2.69)	12.71 (3.57)	<0.001	11.34 (3.04)	12.31 (3.16)	0.216	13.11 (5.55)	19.53 (4.99)	<0.001
Patient care	11.83 (3.13)	12.34 (2.40)	0.286	11.32 (3.11)	13.12 (2.83)	0.02	15.63 (5.51)	22.60 (3.81)	<0.001
Mgmt. and interpersonal	12.35 (3.08)	11.80 (3.39)	0.297	12.61 (3.56)	12.88 (2.92)	0.743	13.77 (5.81)	21.46 (4.48)	<0.001
ERI components^d^									
Effort	16.88 (7.45)	23.15 (5.39)	<0.001	14.76 (7.53)	22.42 (5.82)	<0.001	15.60 (6.31)	24.24 (4.08)	<0.001
Reward	35.14 (12.94)	28.97 (12.11)	0.004	39.68 (12.17)	27.88 (10.86)	<0.001	40.95 (9.24)	35.18 (10.47)	<0.001
Overcommitment	12.58 (4.28)	18.90 (3.96)	<0.001	13.32 (4.66)	19.31 (3.43)	<0.001	14.42 (4.26)	18.15 (3.48)	<0.001

Values are presented as *n* (%) for categorical variables and mean (standard deviation) for continuous variables unless otherwise indicated. *p*-values comparing groups within each dataset were calculated using the chi-square test for categorical variables and the independent *t*-test or Mann–Whitney *U* test for continuous variables. Training and testing sets were derived from the development cohort (*n* = 219); the external validation set is an independent cohort (*n* = 234) from Zhengzhou.

a Data presented as mean (SD).

b Data presented as median [interquartile range] due to non-normal distribution.

c Subscales from the Chinese Nurse Stressor Scale (CNSS).

d Subscales from the Effort-Reward Imbalance (ERI) scale.

### 3.2 Feature selection identifies a parsimonious set of key predictors

**Figure 1:**
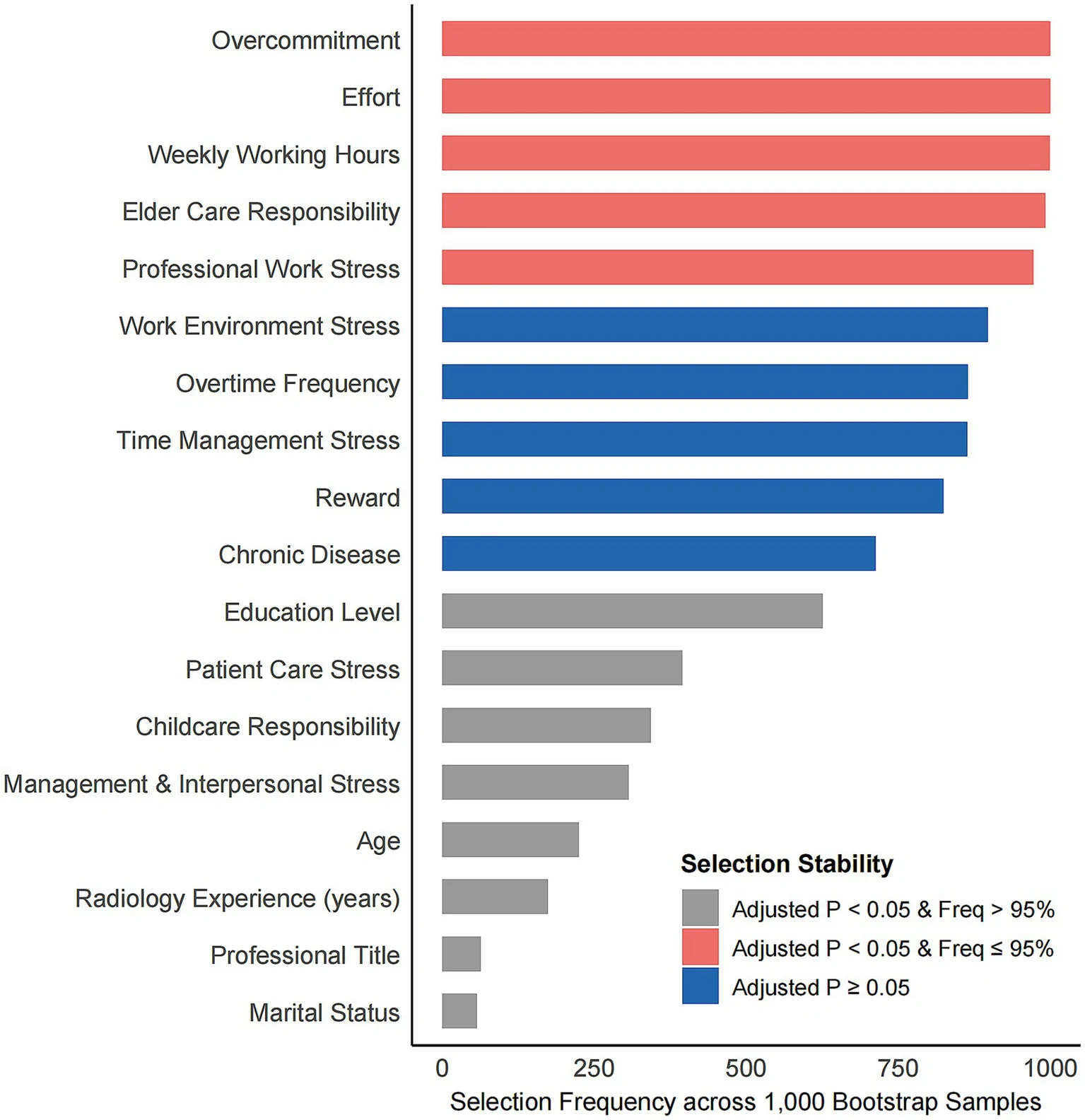
Stability-Driven Feature Selection of Key Predictors. Bootstrap LASSO (*n* = 1,000 resamples) and Permutation Stability Testing (*n* = 1,000 permutations) were performed on the training set. The bar chart shows selection frequency (the number of bootstrap samples in which each predictor was selected). Blue/dark bars indicate predictors that met the stability criterion (selection frequency exceeding the 95th percentile of the null distribution; permutation-adjusted *p* < 0.05). Gray/light bars indicate variables with univariate associations that did not meet the stability threshold.

Stability-driven selection identified five predictors with >95% selection frequency and permutation *p* < 0.05 (Figure 1): Overcommitment, Effort, Weekly Working Hours, Stress from Nursing Profession and Work, and Older adult Care Responsibility. Other variables—including Reward (83.8%), Overtime Frequency (88.3%), and Work Environment Stress (89.9%)—showed univariate associations with burnout but did not meet the stability-selection criterion (selection frequency below the permutation-adjusted significance threshold) and were therefore excluded from the final model to maintain parsimony and robustness.

### 3.3 Model performance: internal testing and external validation

**Figure 2:**
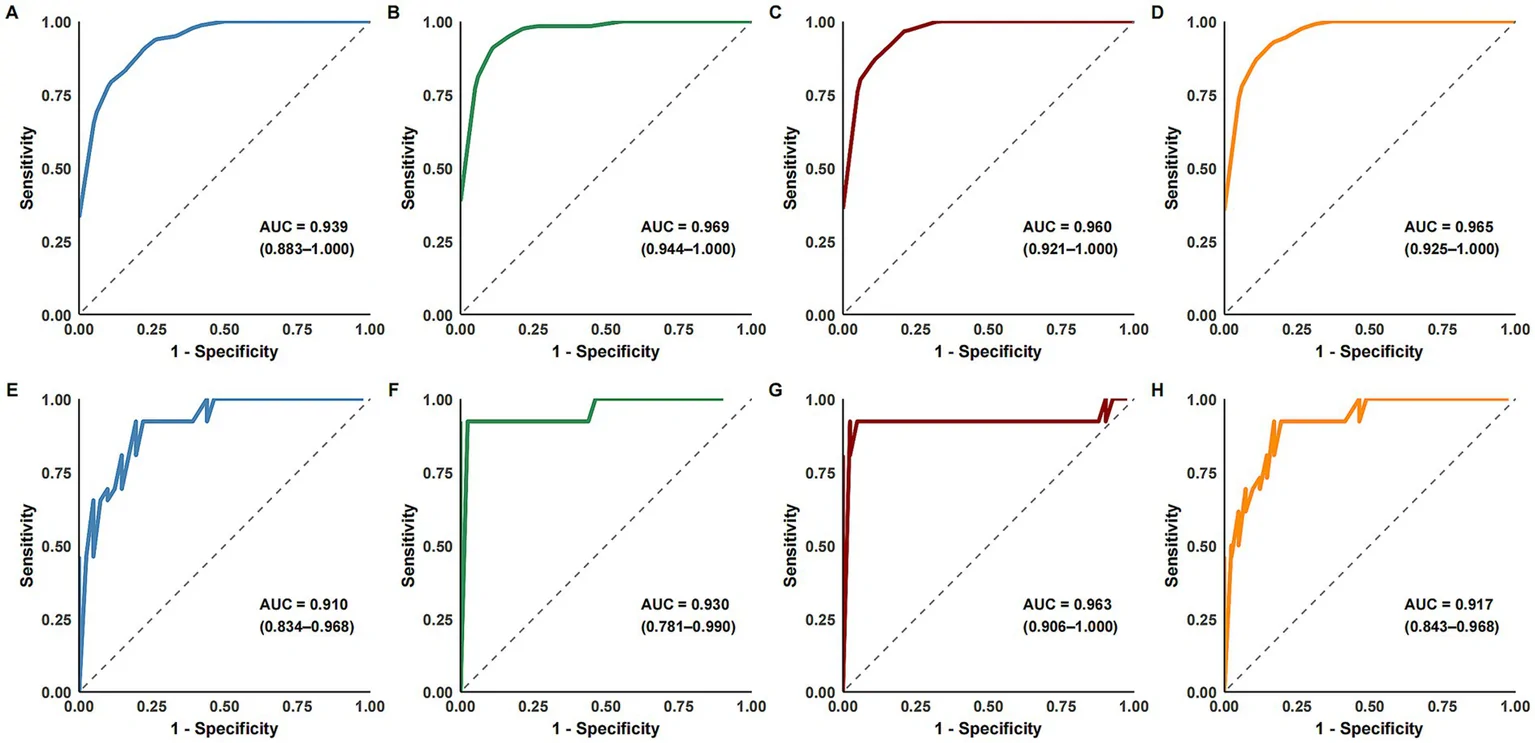
Receiver operating characteristic (ROC) curves for predictive models. Panels A-D present mean ROC curves from 5-fold cross-validation during training. Panels E-H show ROC curves on the independent test set: **(A, E)** Logistic Regression, **(B, F)** Random Forest (RF), **(C, G)** Extreme Gradient Boosting (XGBoost), and **(D, H)** Support Vector Machine (SVM).

During 5-fold cross-validation, all models demonstrated robust and stable performance, as illustrated by the ROC curves in Figures 2A–D. The ensemble models, particularly Random Forest (mean AUC: 0.969 ± 0.026) and XGBoost (mean AUC: 0.960 ± 0.031), consistently showed excellent discriminative potential across the validation folds, indicating successful learning without significant instability (for detailed metrics, see Supplementary Table S3).

On the internal test set, XGBoost emerged as superior (Table 2; Figures 2E–H), achieving AUC 0.963 (95% CI: 0.906–1.000), sensitivity 0.871, specificity 0.953, and Brier score 0.067 (95% CI: 0.031–0.111). This surpassed Logistic Regression (AUC: 0.910), SVM (0.917), and RF (0.900). Sensitivity analyses confirmed robustness (Supplementary Table S4).

**Table 2:** Predictive performance of machine learning models on the independent held-out test set.

Performance metric	Logistic regression	Support vector machine	Random forest	Extreme gradient boosting (XGBoost)
Discrimination				
AUC (95% CI)	0.910 (0.834–0.968)	0.917 (0.843–0.972)	0.930 (0.781–0.990)	**0.963 (0.906–1.000)**
Classification				
F1-Score (95% CI)	0.754 (0.600–0.880)	0.794 (0.666–0.909)	0.890 (0.780–0.977)	**0.871 (0.750–0.962)**
Precision (95% CI)	0.788 (0.609–0.952)	0.830 (0.667–0.964)	1.000 (1.000–1.000)	**0.953 (0.850–1.000)**
Sensitivity (95% CI)	0.729 (0.550–0.897)	0.767 (0.594–0.920)	0.805 (0.640–0.955)	**0.805 (0.640–0.955)**
Specificity (95% CI)	0.876 (0.767–0.974)	0.901 (0.800–0.978)	1.000 (1.000–1.000)	**0.975 (0.915–1.000)**
Calibration				
Brier Score (95% CI)	0.135 (0.080–0.197)	0.115 (0.072–0.165)	0.076 (0.032–0.129)	**0.067 (0.031–0.111)**

Performance metrics were evaluated on the independent test set (*n* = 67, with 26 high-burnout cases). The best-performing model is highlighted in bold. AUC, area under the receiver operating characteristic curve; CI, confidence interval.

### 3.4 Global feature importance and directionality of effects

**Figure 3:**
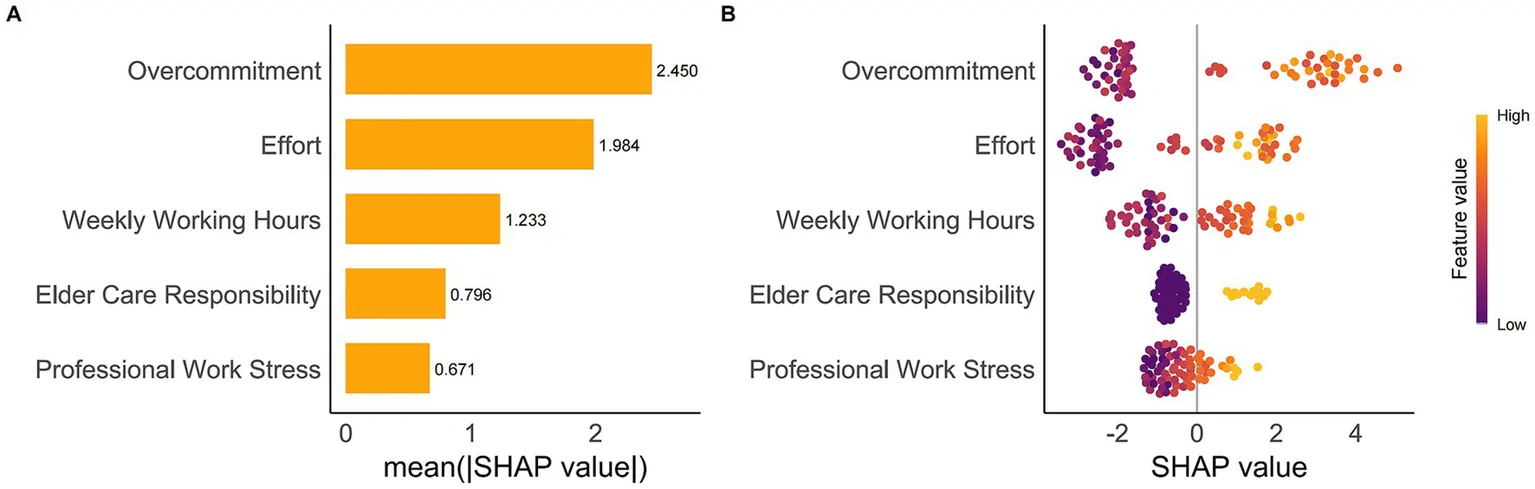
Global feature importance and directionality of effects from SHAP analysis. **(A)**. Mean Absolute SHAP Values. Bar chart showing average impact magnitude, ranked by importance. **(B)**. SHAP Beeswarm Plot. Each point represents an individual nurse’s feature contribution to predicted burnout risk (x-axis: SHAP value). Color (purple: low, yellow/orange: high) indicates local feature value.

SHAP analysis revealed Overcommitment as the most influential predictor (mean |SHAP| = 2.450), followed by Effort (1.984) and Weekly Working Hours (1.233) (Figure 3). Older adult care responsibility and professional work stress showed modest contributions (0.796 and 0.671, respectively). The beeswarm plot (Figure 3A) demonstrates that elevated values consistently increased burnout risk predictions, while professional work stress exhibited heterogeneous effects modulated by contextual factors.

### 3.5 Non-linear relationships and threshold effects revealed by dependence plots

**Figure 4:**
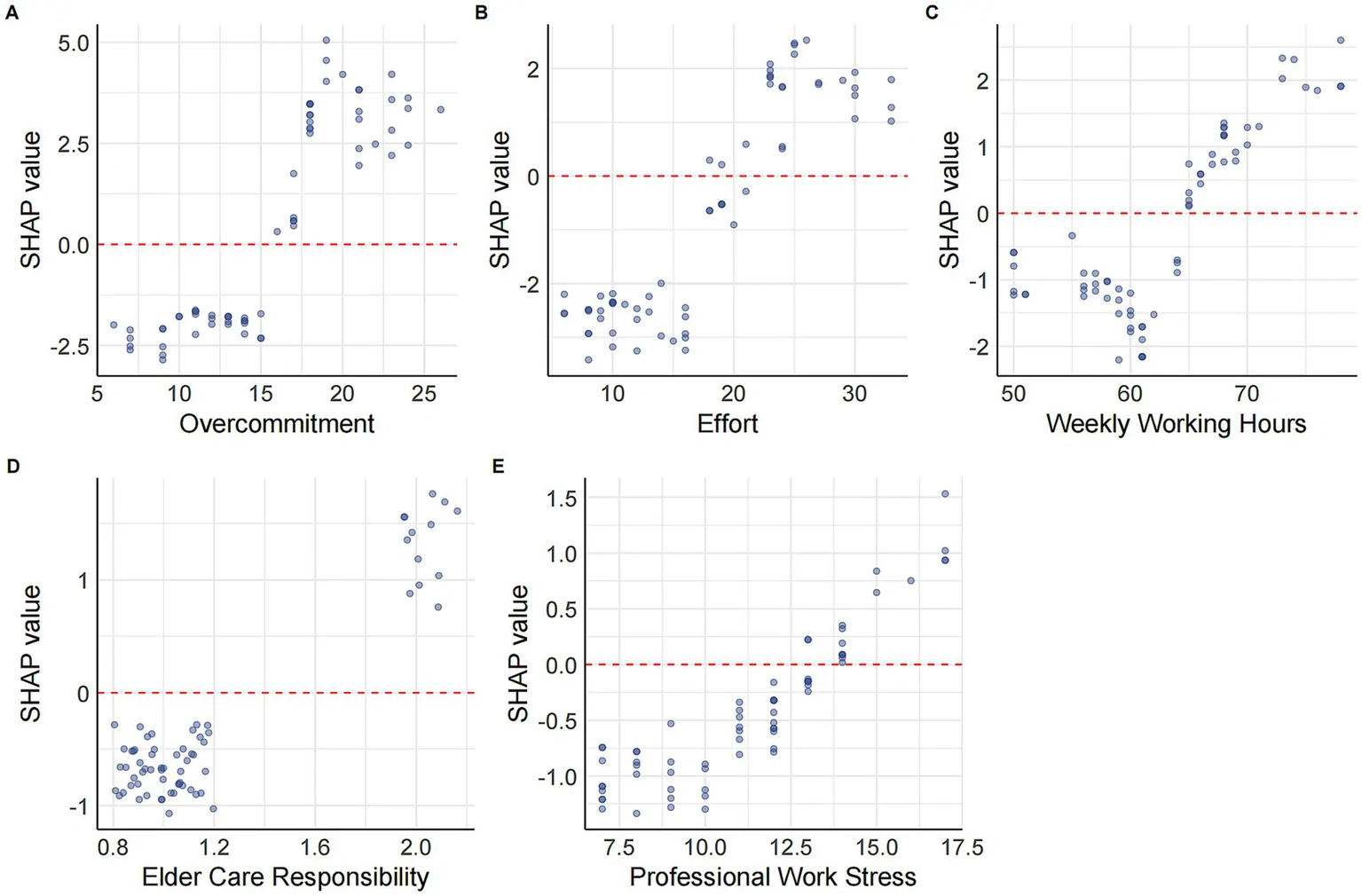
SHAP dependence plots suggesting potential non-linear relationships of key predictors with burnout risk. Derived from the XGBoost model. Panels **(A)–(E)** illustrate the relationship between predictor value (x-axis) and SHAP value (y-axis) for: **(A)** Overcommitment, **(B)** Effort, **(C)** Weekly Working Hours, **(D)** Older adult Care Responsibility, and **(E)** NSS_Professional_Work.

SHAP dependence plots further elucidated the nuanced, non-linear relationships between predictors and burnout risk (Figure 4). For Overcommitment, the most influential feature, a potential inflection point was suggested around a score of 15; below this level, the feature had a negligible impact, but above it, its contribution to burnout risk escalated sharply (Figure 4A). A similar, though less pronounced, suggestive inflection point was observed for Effort around a score of 20 (Figure 4B). The model captured a more linear, dose–response relationship for Weekly Working Hours, with a potential inflection point around 65 h/week, beyond which additional work hours progressively amplified risk (Figure 4C). In contrast, Older adult Care Responsibility acted as a discrete, dichotomous risk factor, with its presence unequivocally increasing predicted burnout risk (Figure 4D). Finally, Professional Work Stress exhibited a monotonic relationship, where its adverse impact grew steadily with increasing stress scores (Figure 4E). Collectively, these granular insights reveal that the risk of burnout does not scale uniformly with stressors; rather, the model identifies specific tipping points beyond which these factors’ detrimental effects accelerate, providing valuable information for defining risk thresholds in clinical practice.

### 3.6 Deconstructing individual predictions with local explanations

**Figure 5:**
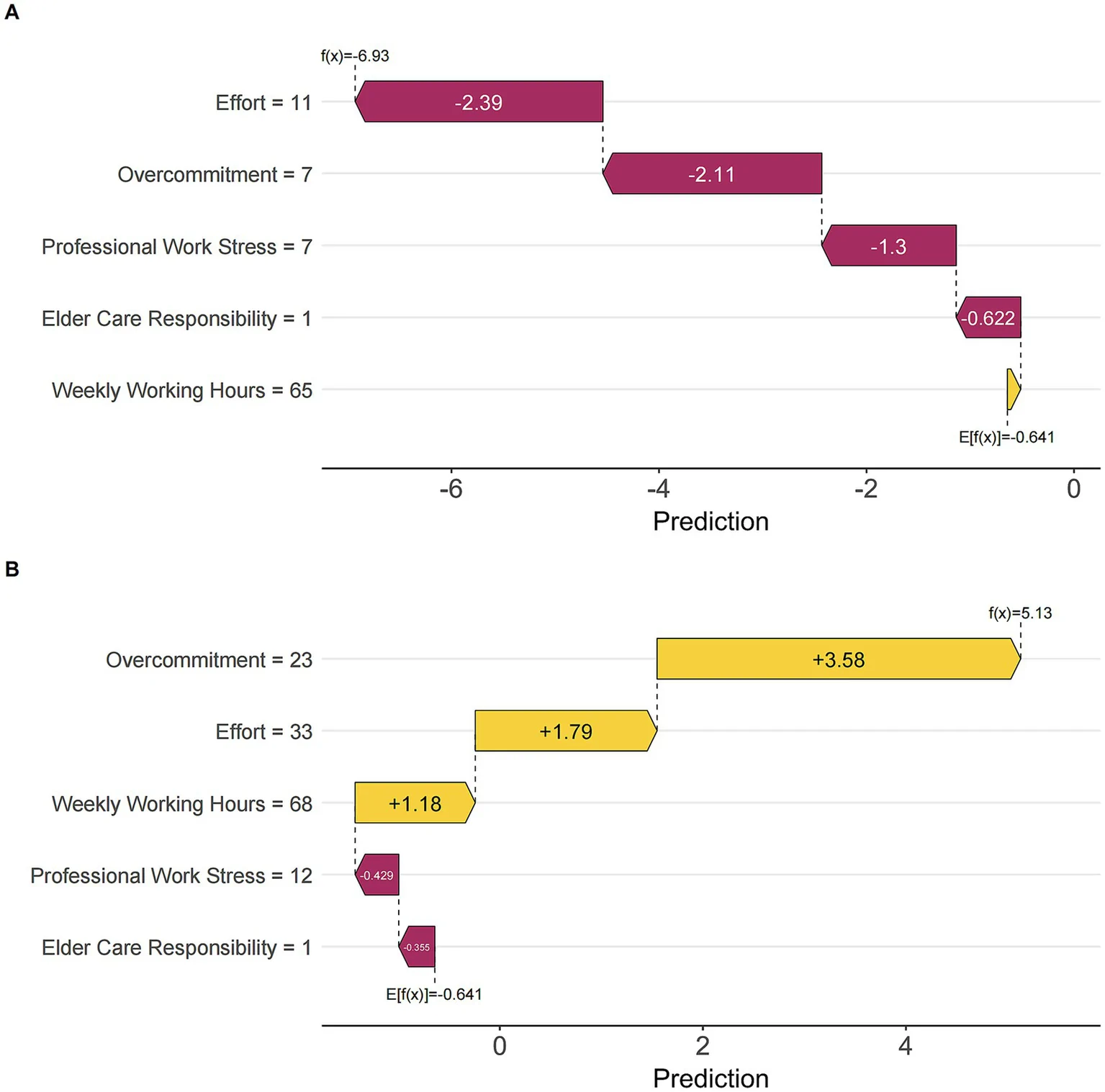
Deconstruction of individual burnout risk predictions with SHAP waterfall plots. **(A)** Waterfall plot for a representative low-risk nurse. **(B)** Waterfall plot for a representative high-risk nurse.

To demonstrate the model’s clinical utility, SHAP waterfall plots were generated to deconstruct the predictions for prototypical cases (Figure 5). For a representative low-risk nurse (Figure 5A), the prediction was dominated by protective factors: low levels of Effort (SHAP = −2.39) and Overcommitment (SHAP = −2.11), along with low professional stress (SHAP = −1.3). These contributions effectively neutralized the risk associated with working near the 65-h threshold, resulting in a strongly negative final prediction.

Conversely, for a representative high-risk nurse (Figure 5B), the prediction was primarily driven by exceptionally high scores in Overcommitment (SHAP = +3.58) and Effort (SHAP = +1.79), compounded by long weekly work hours (SHAP = +1.18). These three factors collectively overwhelmed the minor protective effects of moderate professional stress and the absence of older adult care duties, culminating in a high final prediction score.

### 3.7 Robustness and generalizability: external validation and clinical utility

**Figure 6:**
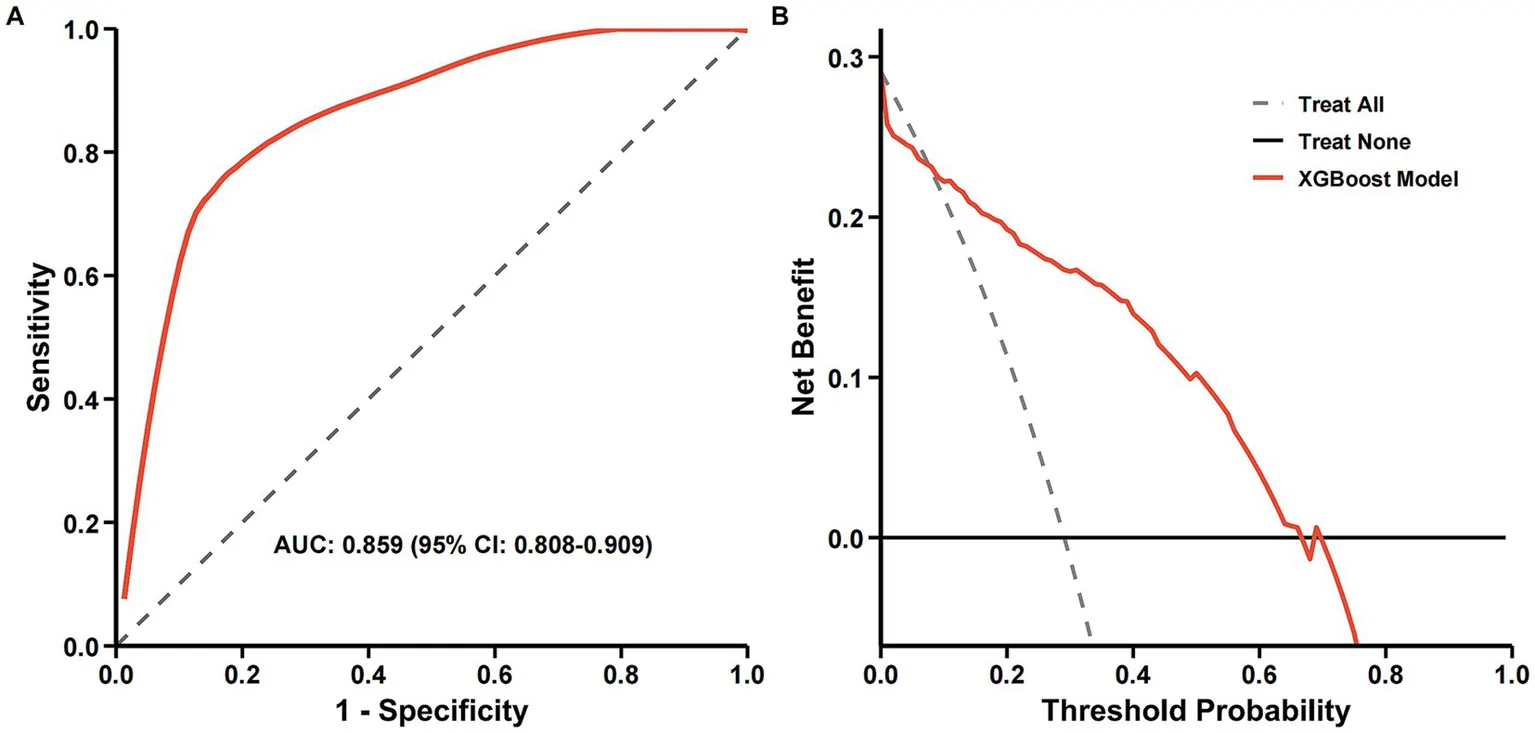
Performance evaluation in the independent external validation cohort. **(A)** Receiver Operating Characteristic (ROC) curve of the finalized XGBoost model applied to the external validation set. **(B)** Decision Curve Analysis (DCA) assessing the clinical utility of the model.

To evaluate the reliability of the finalized XGBoost model in a real-world clinical context distinct from the derivation setting, we applied only the XGBoost model to the external validation cohort (*n* = 234) without any re-calibration; the four-model comparison was reserved for the internal test set (Table 2). Despite the differing burnout prevalence and demographic shifts described in the baseline characteristics, the model demonstrated strong discriminative power, achieving an AUC of 0.859 (95% CI: 0.808–0.909) (Figure 6A). The model also demonstrated satisfactory overall performance, with an accuracy of 0.786 (95% CI: 0.729–0.838), sensitivity of 0.735 (95% CI: 0.618–0.838), specificity of 0.811 (95% CI: 0.745–0.867), precision of 0.617 (95% CI: 0.504–0.722), and F1-score of 0.671 (95% CI: 0.580–0.754). The Brier score was 0.165 (95% CI: 0.135–0.200). Calibration assessment revealed an intercept of −0.12 and a slope of 0.94, both close to ideal values (0 and 1, respectively), indicating good alignment between predicted probabilities and observed outcomes (Supplementary Table S5). The calibration performance across deciles of predicted risk is presented in Supplementary Table S6.

Beyond discrimination accuracy, Decision Curve Analysis (DCA) was performed to assess the model’s clinical utility (Figure 6B). The analysis revealed that the XGBoost model (red line) provided a higher net benefit than the default strategies of “treating all” nurses (gray dashed line) or “treating none” (black solid line) across a wide range of threshold probabilities, approximately from 5 to 65%.

Confusion matrices for the XGBoost model at the 0.5 probability threshold in both the internal test set and external validation cohort are provided in Supplementary Table S7.

## 4 Discussion

This study represents a methodological advancement in occupational health research by developing and validating an interpretable machine learning framework to predict individual burnout risk among radiology nurses. Our XGBoost model not only achieved strong discriminative performance in the development cohort (AUC: 0.963) but also demonstrated robust generalizability in an independent external validation cohort (AUC: 0.859). By distilling five critical predictors from 22 candidates and revealing non-linear dose–response relationships—including a pivotal 65-h weekly workload threshold and an overcommitment score inflection point at 15—this framework transcends the limitations of traditional epidemiological approaches that describe population-level associations but fail to capture individual-level risk trajectories.

### 4.1 Contextualization within existing evidence

Our finding of high burnout prevalence (38.8% in development, 29.1% in external validation) substantially exceeds the 11.23% global pooled prevalence reported in Woo et al.’s multinational meta-analysis (19) and even surpasses the 28.1% rate documented among Chinese mental health nurses (20). This disparity underscores that radiology nurses constitute an ultra-high-risk subpopulation, facing compounding pressures from radiation safety protocols, rapid technological adaptation, and the “hidden workload” of on-call duties and administrative tasks that extend actual work hours far beyond scheduled shifts (6). Critically, our machine learning approach corroborates prior findings while adding precision: whereas Shah et al. (21) identified work demands as correlates of burnout using logistic regression (OR: 1.02 per additional hour), our SHAP dependence plots revealed that this relationship is profoundly non-linear, with a sharp inflection at 65 h per week—beyond which marginal increases in workload precipitate disproportionate escalations in burnout risk.

The primacy of psychosocial factors over demographics in our model aligns with established literature. Yuan et al. (22) demonstrated that effort-reward imbalance (ERI) was the strongest predictor of burnout among Chinese healthcare workers, while Li et al.’s JAMA Network Open meta-analysis (7) confirmed that modifiable work characteristics exert far greater influence than immutable personal attributes. Our decision to decompose the ERI construct into separate effort, reward, and overcommitment components—rather than employing the traditional ERI ratio—proved superior in sensitivity analyses and resonates with findings from a prior mediation study (6), which demonstrated that ERI mediated 47.47% of the work stress-burnout pathway. This convergence across methodologies (structural equation modeling for explanation, machine learning for prediction) provides robust evidence that overcommitment (mean |SHAP| = 2.450) and effort (mean |SHAP| = 1.984) are the dominant drivers of burnout, operating through mechanisms distinct from their ratio-based formulation.

### 4.2 Novel contributions: non-linear thresholds and interpretable prediction

Perhaps the most consequential contribution lies in suggesting potential risk thresholds invisible to linear models. The potential inflection point at approximately 65 weekly work hours, as suggested by the SHAP dependence plot, is consistent with the “allostatic load” theory (23), wherein chronic stress induces cumulative physiological wear with a critical tipping point beyond which homeostatic mechanisms fail. This finding challenges conventional linear assumptions and may have translational relevance. Rather than generic workload reduction policies, our data suggest that interventions such as mandatory hour caps could be considered—analogous to the European Working Time Directive, which has been associated with reduced burnout in countries with strict enforcement. However, this threshold remains hypothesis-generating and requires prospective validation before policy adoption (24). Similarly, the overcommitment threshold (score ≈15) aligns with Hobfoll’s conservation of resources theory (25), suggesting that below this level, nurses maintain precarious equilibrium, but beyond it, resource depletion spirals into exhaustion. Crucially, beyond statistical metrics, our Decision Curve Analysis (DCA) in the external cohort confirmed the model’s utility for occupational health decision-making. By showing a positive net benefit across a broad range of threshold probabilities (5–65%), we demonstrate that using this algorithmic screening approach is superior to ‘one-size-fits-all’ wellness programs. This translates to more efficient resource allocation: nurse managers can direct intensive support specifically to those truly at risk, avoiding the waste of applying generic interventions to the low-risk majority.

Our integration of SHAP analysis directly addresses the “black box” problem that has hindered clinical machine learning adoption. While recent studies have applied gradient boosting to predict physician burnout from electronic health record use (26), few have provided granular, individual-level explanations. The SHAP waterfall plots (Figure 5) demonstrate how our model can generate personalized risk profiles—revealing not just who is at risk but why—thereby enabling targeted interventions. For instance, a nurse flagged as high-risk due to elevated overcommitment and approaching the 65-h threshold could receive immediate workload redistribution and counseling, whereas population-wide wellness programs may dilute impact by treating all nurses uniformly (27).

Our identification of older adult care responsibility as a discrete, dichotomous risk factor (mean |SHAP| = 0.796) adds a novel dimension often overlooked in nursing burnout research. This finding aligns with work–family conflict theory (28) and has particular salience in China’s rapidly aging society, where the proportion of citizens aged ≥60 is projected to exceed 30% by 2040 (29). The psychological burden of role conflict—not simply absolute caregiving workload—appears operative, suggesting that family-supportive policies (flexible scheduling, older adult care resources) should be integral to burnout prevention strategies, not peripheral benefits.

It is important to note that SHAP dependence plots are exploratory tools for visualizing patterns in model predictions rather than formal threshold estimation methods. The potential inflection points identified in our analysis should therefore be interpreted as data-driven hypotheses requiring confirmation through dedicated change-point detection methods in independent datasets.

### 4.3 Methodological rigor and comparative performance

Our stability-driven feature selection protocol, combining bootstrapped LASSO with permutation testing, represents a significant advancement over conventional stepwise regression prone to overfitting (18). By requiring predictors to demonstrate both high selection frequency (>95%) and statistical significance against a null distribution, we ensured model parsimony without sacrificing predictive power. The XGBoost algorithm’s superior performance (AUC: 0.963) compared to prior machine learning studies in nursing burnout (30–32), likely stems from this rigorous feature engineering coupled with hyperparameter optimization via 5-fold cross-validation. A distinctive strength of our study, often lacking in similar ML applications, is the rigorous external validation in a temporally and geographically independent cohort. While many predictive models suffer from ‘optimism bias’ and degrade significantly when applied to new data, our model maintained high discrimination (AUC > 0.85) without retraining. This suggests that the feature selection process successfully filtered out noise and captured the genuine, stable signals of burnout risk.

Critically, our multi-layered interpretability framework—encompassing global importance rankings, dependence plots revealing thresholds, and waterfall plots deconstructing individual predictions—transforms the model from a prediction engine into a decision-support tool. This addresses the gap identified in Beam and Kohane’s systematic review (33), which found that only 12% of clinical machine learning studies provided model explanations. Our approach demonstrates that interpretable AI can simultaneously achieve high predictive accuracy and clinical actionability.

### 4.4 Limitations and future directions

Our study has several limitations. First, despite the robust temporal validation with independent participants (1.5-year interval), albeit with partial geographic overlap at one site, the underlying data relied on cross-sectional surveys, which precludes definitive causal inference regarding the directional relationship between stressors and burnout. Second, the development cohort was modest in size (85 burnout events, 38.8% prevalence). Overfitting risks were mitigated through strict adherence to the 15 EPV rule (final five predictors) and XGBoost regularization. While data-driven selection from 22 candidates may bias the internal AUC (0.963) upward, the robust external validation AUC (0.859, without recalibration) supports generalizability. Larger studies are needed for further validation. Third, while our multi-center design covered seven major hospitals across temporally independent regions (including North, Southwest, and Central China), the distinct focus on Grade-A tertiary hospitals restricts generalizability to secondary or community-based healthcare facilities, where resource constraints and workflow dynamics may differ. Fourth, the internal test set comprised only 67 participants with 26 burnout events, resulting in wide confidence intervals for several performance metrics. While the test set size was appropriate for preliminary internal validation, the model’s generalizability is more reliably estimated by the larger external validation cohort (*n* = 234). Fifth, the exclusion of rotating, visiting, and trainee nurses—a decision made to ensure feature stability—may have yielded conservative prevalence estimates, as this “floating workforce” often endures greater job insecurity and reward deprivation. Sixth, reliance on self-reported psychometric instruments, though validated, introduces inevitable response bias. Future iterations of the model would benefit from integrating objective physiological markers (e.g., heart rate variability via wearable biosensors) or operational data (e.g., electronic health record logs) to reduce subjectivity. Seventh, while our SHAP analysis suggested potential non-linear risk thresholds (e.g., approximately 65 work hours/week), these findings are exploratory by nature. SHAP dependence plots are designed for model interpretation, not for formal threshold estimation. Formal validation using change-point detection methods in independent prospective cohorts is needed before these thresholds can be translated into clinical practice guidelines or regulatory policies. Eighth, the study was not designed to compare burnout risk across radiology subspecialties (e.g., radiotherapy vs. interventional radiology vs. diagnostic imaging). Future studies with larger sample sizes within each subspecialty are needed to explore potential differences. Finally, while our external validation demonstrated satisfactory calibration without re-calibration, we acknowledge that in future clinical implementation at new sites, simple intercept recalibration could further improve local calibration performance. This should be considered when deploying the model to different clinical settings.

Looking forward, research should pivot from model validation to implementation science. Since the model has demonstrated discriminatory power and clinical net benefit in retrospective cohorts, the next logical step is a pragmatic randomized controlled trial (RCT). Such a trial should compare the efficacy of AI-guided, precision interventions—triggered by our XGBoost thresholds (e.g., >65 work hours, >15 overcommitment)—against standard “wellness programs” in reducing actual burnout incidence. Additionally, longitudinal surveillance is needed to determine the temporal window of prediction, assessing how far in advance the model can flag deterioration before clinical burnout manifests.

## 5 Conclusion

This study develops and validates an interpretable machine learning framework that successfully transcends traditional epidemiological descriptions, offering a precision prevention approach to occupational burnout prediction. Validated across independent temporal settings (with a 1.5-year interval) and across multiple centers (with partial institutional overlap), our XGBoost-SHAP model demonstrates robust generalizability, proving that core risk signals—specifically the potential non-linear inflection points of workload and overcommitment suggested by the SHAP analysis—remain stable metrics across different hospital environments and timeframes. By deconstructing the “black box” of prediction into distinguishing individual risk profiles, this framework enables a shift from reactive crisis management to proactive, data-driven prevention. For radiology nurses—who navigate the unique intersection of radiation safety, technological complexity, and hidden workloads—this approach provides a scientifically grounded tool to guide resource allocation and intervention. As healthcare systems globally confront workforce attrition, the integration of such validated, transparent, and high-utility predictive analytics into occupational health governance represents not merely a technical advancement but also an important consideration for occupational health policy.

## Data Availability

The raw data supporting the conclusions of this article will be made available by the authors, without undue reservation.

## Footnotes

1
www.mindgarden.com
