# Supplementary material for: Predicting burnout in radiology nurses: an interpretable machine learning model developed and externally validated in a multi-center study
Source: Front Public Health. 2026 Aug 11;14:1914779. doi: 10.3389/fpubh.2026.1914779 (PMC13503554; doi:10.3389/fpubh.2026.1914779)
Supplement: Supplementary file 1 [file Table_1.docx]

**Supplementary Materials**

****Supplementary Materials****

****Supplementary Table S1.** Hyperparameter search spaces and optimal values for each of the four machine learning algorithms (Logistic Regression, Support Vector Machine, Random Forest, and Extreme Gradient Boosting).**

****Supplementary Table S2.** Sensitivity analysis: XGBoost performance using an alternative burnout definition based on dimensional cutoffs (emotional exhaustion > 12, depersonalization > 7, reduced personal accomplishment > 18; high burnout defined as ≥ 2 dimensions). Results are shown for both the internal test set and external validation cohort.**

****Supplementary Table S3.** Model performance metrics (AUC, Sensitivity, Specificity, Precision, F1-Score, Brier Score) for four machine learning models (Logistic Regression, Support Vector Machine, Random Forest, and Extreme Gradient Boosting) during 5-fold cross-validation on the training set. Results are reported as Mean ± Standard Deviation.**

****Supplementary Table S4.** Performance of the primary XGBoost model in sensitivity analyses, comparing it with variations that included the Effort-Reward Imbalance (ERI) Ratio or excluded chronic diseases as a predictor. Metrics (AUC, F1-Score, Precision, Sensitivity, Specificity, Brier Score) with 95% Confidence Intervals are provided for the independent test set.**

****Supplementary Table S5.** External validation performance metrics of the XGBoost model, including discrimination (AUC), classification accuracy (accuracy, sensitivity, specificity, precision, F1-score), and calibration metrics (Brier score, calibration intercept, calibration slope). The model was applied to the external validation cohort (n=234) without any re-training or re-calibration.**

****Supplementary Table S6.** Calibration of the XGBoost model in the external validation cohort, presented by deciles of predicted risk, showing mean predicted probability and observed proportion of burnout for each decile.**

****Supplementary Table S7.** Confusion matrices for the XGBoost model at the 0.5 probability threshold in the internal test set (n=67) and external validation cohort (n=234).**

**Supplementary Table S1.** Hyperparameter Search Spaces and Optimal Values for Each Algorithm

| Algorithm | Hyperparameter | Search Space / Values Tested | Optimal Value |
| --- | --- | --- | --- |
| **Logistic Regression** | C (inverse regularization strength) | 0.001, 0.01, 0.1, 1, 10, 100 | 1 |
|  | penalty | L1, L2 | L2 |
|  | solver | liblinear, saga | liblinear |
| **SVM (RBF kernel)** | C (regularization) | 0.1, 1, 10, 100 | 10 |
|  | gamma (kernel coefficient) | 0.001, 0.01, 0.1, 1 | 0.01 |
| **Random Forest** | n_estimators | 50, 100, 200, 500 | 200 |
|  | max_depth | 3, 5, 7, 10, None | 7 |
|  | min_samples_split | 2, 5, 10 | 5 |
|  | min_samples_leaf | 1, 2, 4 | 2 |
|  | max_features | sqrt, log2, None | sqrt |
| **XGBoost** | n_estimators | 50, 100, 200, 500 | 200 |
|  | max_depth | 3, 5, 7, 10 | 5 |
|  | learning_rate | 0.01, 0.05, 0.1, 0.3 | 0.1 |
|  | subsample | 0.6, 0.8, 1.0 | 0.8 |
|  | colsample_bytree | 0.6, 0.8, 1.0 | 0.8 |
|  | min_child_weight | 1, 3, 5 | 3 |
|  | scale_pos_weight | ratio of negative/positive samples | 1.58 |
|  | gamma | 0, 0.1, 0.5, 1 | 0.1 |

**Table Note:** Hyperparameter optimization was performed via 5-fold cross-validation on the training set (n=152), guided by maximizing the mean AUC. scale_pos_weight for XGBoost was calculated as the ratio of negative to positive samples in the training set: (152 − 59) / 59 ≈ 1.58.

**Supplementary Table S2.** Sensitivity Analysis: XGBoost Performance Using Alternative Burnout Definition (Dimensional Cutoffs)

| Performance Metric | Internal Test Set (n = 67) | External Validation (n = 234) |
| --- | --- | --- |
| AUC (95% CI) | 0.951 (0.892–0.990) | 0.847 (0.795–0.895) |
| Accuracy (95% CI) | 0.896 (0.810–0.955) | 0.778 (0.720–0.830) |
| Sensitivity (95% CI) | 0.852 (0.700–0.955) | 0.721 (0.610–0.820) |
| Specificity (95% CI) | 0.925 (0.810–0.985) | 0.802 (0.730–0.865) |
| F1-Score (95% CI) | 0.861 (0.740–0.945) | 0.659 (0.570–0.742) |

**Table Note:** Alternative burnout definition based on dimensional cutoffs: emotional exhaustion > 12, depersonalization > 7, reduced personal accomplishment > 18; high burnout defined as meeting criteria on ≥ 2 dimensions. These cutoffs were derived from the Chinese version of the MBI-GS validated in healthcare worker populations. All metrics were calculated using the default 0.5 probability threshold.

### ****Supplementary Table S3.** Model Performance during 5-Fold Cross-Validation on the Training Set**

| **Performance Metric** | **Logistic Regression** | **Support Vector Machine** | **Random Forest** | **Extreme Gradient Boosting (XGBoost)** |
| --- | --- | --- | --- | --- |
| ****AUC**** | 0.939 ± 0.059 | 0.965 ± 0.018 | ****0.969 ± 0.026**** | 0.960 ± 0.031 |
| ****Sensitivity**** | 0.883 ± 0.173 | 0.809 ± 0.174 | 0.848 ± 0.108 | ****0.850 ± 0.091**** |
| ****Specificity**** | 0.870 ± 0.101 | 0.849 ± 0.121 | 0.923 ± 0.115 | ****0.968 ± 0.029**** |
| ****Precision**** | 0.819 ± 0.116 | 0.810 ± 0.126 | 0.903 ± 0.128 | ****0.945 ± 0.050**** |
| ****F1-Score**** | 0.842 ± 0.120 | 0.787 ± 0.049 | 0.865 ± 0.072 | ****0.892 ± 0.052**** |
| ****Brier Score**** | 0.098 ± 0.058 | 0.095 ± 0.017 | 0.071 ± 0.039 | ****0.070 ± 0.030**** |

Values are reported as Mean ± Standard Deviation across the 5 folds of the cross-validation procedure conducted on the training set (n=152). Models were trained using the five selected predictors. The best-performing model in each metric is highlighted in ****bold****. AUC, Area Under the Receiver Operating Characteristic Curve.

**Supplementary Table S4. Performance of XGBoost Model in Sensitivity Analyses**

| **Performance Metric** | **Primary XGBoost Model**  **(for reference)** | **XGBoost with**  **ERI Ratio** | **XGBoost Excluding**  **Chronic Diseases** |
| --- | --- | --- | --- |
| **Discrimination** |  |  |  |
| AUC (95% CI) | 0.963 (0.906–1.000) | 0.945 (0.880–0.992) | 0.961 (0.902–0.998) |
| **Classification** |  |  |  |
| F1-Score (95% CI) | 0.871 (0.750–0.962) | 0.850 (0.730–0.950) | 0.865 (0.740–0.958) |
| Precision (95% CI) | 0.953 (0.850–1.000) | 0.930 (0.830–0.990) | 0.945 (0.840–0.995) |
| Sensitivity (95% CI) | 0.805 (0.640–0.955) | 0.790 (0.620–0.940) | 0.800 (0.630–0.950) |
| Specificity (95% CI) | 0.975 (0.915–1.000) | 0.960 (0.890–1.000) | 0.970 (0.900–1.000) |
| **Calibration** |  |  |  |
| Brier Score (95% CI) | 0.067 (0.031–0.111) | 0.085 (0.040–0.130) | 0.070 (0.035–0.115) |

**Table Notes:**
Performance metrics were evaluated on the independent test set. The “Primary XGBoost Model” column is included for direct comparison with the main results in Table 2. AUC, Area Under the Receiver Operating Characteristic Curve; CI, Confidence Interval; ERI, Effort-Reward Imbalance.

**Supplementary Table S5.** External Validation Performance of the XGBoost Model (n = 234)

| Performance Metric | Value |
| --- | --- |
| **Discrimination** |  |
| AUC (95% CI) | 0.859 (0.808 – 0.909) |
| **Classification (at threshold 0.5)** |  |
| Accuracy (95% CI) | 0.786 (0.729 – 0.838) |
| Sensitivity (95% CI) | 0.735 (0.618 – 0.838) |
| Specificity (95% CI) | 0.811 (0.745 – 0.867) |
| Precision (95% CI) | 0.617 (0.504 – 0.722) |
| F1-Score (95% CI) | 0.671 (0.580 – 0.754) |
| **Calibration** |  |
| Brier Score (95% CI) | 0.165 (0.135 – 0.200) |
| Calibration Intercept | –0.12 |
| Calibration Slope | 0.94 |

**Table Note:** The XGBoost model was applied to the external validation cohort (n=234) without any re-training or re-calibration. Predicted probabilities were generated using the model's native logistic transformation with the default 0.5 classification threshold. All 95% confidence intervals were derived from 2,000 participant-level bootstrap replicates. AUC, Area Under the Receiver Operating Characteristic Curve; CI, Confidence Interval.

**Supplementary Table S6.** Calibration of the XGBoost Model in the External Validation Cohort (by Deciles of Predicted Risk)

| Decile | Predicted Risk Range | n | Mean Predicted Probability | Observed Proportion (Burnout) |
| --- | --- | --- | --- | --- |
| 1 | 0 – 10% | 24 | 0.06 | 0.04 |
| 2 | 10 – 20% | 23 | 0.14 | 0.09 |
| 3 | 20 – 30% | 24 | 0.25 | 0.21 |
| 4 | 30 – 40% | 23 | 0.35 | 0.33 |
| 5 | 40 – 50% | 24 | 0.44 | 0.46 |
| 6 | 50 – 60% | 23 | 0.55 | 0.58 |
| 7 | 60 – 70% | 24 | 0.64 | 0.68 |
| 8 | 70 – 80% | 23 | 0.75 | 0.74 |
| 9 | 80 – 90% | 24 | 0.84 | 0.79 |
| 10 | 90 – 100% | 22 | 0.93 | 0.86 |

**Table Note:** The external validation cohort (n=234) was divided into 10 equal-sized groups (deciles) based on the XGBoost model's predicted burnout risk. For each decile, the mean predicted probability is compared with the observed proportion of participants who were classified as having high burnout (MBI-GS ≥ 3.5). In a perfectly calibrated model, the observed proportion would equal the mean predicted probability within each decile. Values shown are based on the model's raw predictions without any post-hoc probability calibration.

**Supplementary Table S7.** Confusion Matrices for the XGBoost Model at the 0.5 Probability Threshold

**(a) Internal Test Set (n = 67)**

|  | Predicted: Non-Burnout | Predicted: High Burnout | Total |
| --- | --- | --- | --- |
| **Actual: Non-Burnout** | 40 | 1 | 41 |
| **Actual: High Burnout** | 5 | 21 | 26 |
| **Total** | 45 | 22 | 67 |

**(b) External Validation Cohort (n = 234)**

|  | Predicted: Non-Burnout | Predicted: High Burnout | Total |
| --- | --- | --- | --- |
| **Actual: Non-Burnout** | 135 | 31 | 166 |
| **Actual: High Burnout** | 18 | 50 | 68 |
| **Total** | 153 | 81 | 234 |

**Table Note:** Classification metrics reported in Table 2 (internal test set) and Supplementary Table S3 (external validation) were derived from these confusion matrices using the default probability threshold of 0.5
